# Supplementary material for: Development and Application of Computerized Risk Registry and Management Tool Based on FMEA and FRACAS for Total Testing Process
Source: Medicina (Kaunas). 2021 May 11;57(5):477. doi: 10.3390/medicina57050477 (PMC8151538; doi:10.3390/medicina57050477)
Supplement: Supplementary file 1 [file medicina-57-00477-s001.zip › medicina-1152938-supplementary.pdf]

Table S1. Example of risk management cases of the study.

								Before action				Post action			
**No.**	**Stage***	**Failure mode**	**Effect**	**Cause**	**Plan**	**Action**	**Result**	**S**	**F**	**D**	**R**	**S**	**F**	**D**	**R**
1	2	It is requested to test tests that cannot be done with BF samples	1. Inaccurate results reported.<br>2. When inquiring cumulative results, BF results overlap with blood test results, causing confusion.	1. In CPOE, samples of any kind are allowed to be mapped to the any test item.	1. Staff training in the reception unit (to check the sample type)<br>2. Staff training in testing unit (to check the sample type)<br>3. Modify CPOE to prescribe only specified samples by test.	1. Done<br>2. Done<br>3. Can be implemented in CPOE replacement years from now	Risk management action is done.	1	3	3	9	1	3	3	9
2	3	Missing urine sample in creatine clearance test	1. Delete reported serum result<br>2. Retest using resampled urine and serum sample<br>3. Unnecessary reagent consumption<br>4. Result delayed	Lack of concentration in nurses	1. Nurse training<br>2. Staff training in the reception unit (to check the sample pair)	1. Done<br>2. Done	Monitoring should be continued.	3	4	5	60	3	4	3	36
3	3	Collect random urine for 24-hour urine prescription	1. Invalid result reported<br>2. Resampling needed	Lack of concentration in nurses	1. Nurse training<br>2. Staff training in the reception unit (to check the sample type)	1. Done<br>2. Done	Monitoring should be continued.	3	3	3	27	3	3	2	18
4	4	Barcode reading error	1. Result delayed	Bad barcode print status	1. Nurse training<br>2. Staff training in the reception unit (to check the sample quality)	1. Done<br>2. Done	Monitoring should be continued.	1	5	3	15	1	5	3	15

5	4	1. Two different reagents interchanged and incorrectly installed<br>2. Did not prime after reagent installation	Calibration and QC passed because there was a reagent left in the line, and the patient results were incorrectly measured afterwards.	1. Both reagent bins look the same<br>2. Not familiar with the instructions for reagent replacement	1. Attach the warning sign to the location of the reagent mounting.<br>2. Staff training to re-check the instructions<br>3. Provide a summary of the instruction around the work place<br>4. Requesting design change to manufacturer	1. Done<br>2. Done<br>3. Done<br>4. Requested, but not yet modified	Monitoring should be continued.	5	2	2	20	5	2	1	10
6	5	Incorrect retesting results reported with existing sample during resampling	1. Incorrect retesting results reported<br>2. Incorrect treatment applied	1. Request to resample without reporting results<br>2. The reception was canceled only by computer. Existing sample was not actually disposed	1. All CVR results will be re-examined and the result will be entered.<br>2. Do not leave results blank for reasons of resampling<br>3. Take the sample in your hand, cancel the computer application, and discard it	1. Training completed<br>2. Instruction is modified	Risk management action is done.	3	3	3	27	3	3	1	9
7	5	Instrument stopped during testing due to insufficient sample volume	1. Result delayed<br>2. Resampling needed	Insufficient sample volume	1. Blood collector training<br>2. Staff training<br>3. Sample volume check during preanalytic phase	1. Done<br>2. Done<br>3. Done	Monitoring should be continued.	3	4	2	24	3	4	1	12
8	6	Reported incorrect results without checking Flag sign	1. Invalid result reported<br>2. Retesting needed	Lack of concentration in staff	1. Highlight the flag so that it can be seen clearly visible<br>2. Staff training to re-check the instructions<br>3. Provide a summary of the instruction	1. Done<br>2. Done<br>3. Done	Risk management action is done.	3	3	2	18	3	2	1	6

					around the work place										
9	6	Result is reported without re-examination even though delta/panic verification has been detected and aspiration error occurred	1. Invalid result reported<br>2. Retesting needed	1. Foreign substances (fibrinogen, micro clots) are present in serum<br>2.Too many results that fail delta and panic verification.<br>3. Subjective judgment of workers whether to re-examine or not	1.Staff training in sampling unit<br>- Mix samples six times after collecting blood<br>- Centrifugal separation after 10 to 15 minutes of blood collection<br>2. Staff training in testing unit<br>- Test after checking for foreign substances with eyes<br>3. The delta criteria for the interface and LIS must be the same<br>4. Modify delta criteria, which can reduce retesting rate<br>5. Automatic re-examination of all items in case of aspiration error occurs.	1. Done<br>2. Done<br>3. Done<br>4. In the process of verifying data<br>5. Done	Monitoring should be continued.	3	3	2	18	3	2	2	12
10	6	Results reported as blank	1. Invalid result reported<br>2. Retesting needed	The result was not properly verified	Check again before reporting results	Done	Monitoring should be continued.	3	3	2	18	3	2	2	12

Abbreviations: BF, body fluid; CPOE, Computerized physician order entry; CVR, critical value reporting; D, detection capability; F, frequency; S, severity; QC, quality control; R; RPN (risk priority number). *The stage of failure mode was divided into six according to the testing sequence: 1, patient registration; 2, test request; 3, preanalytical (pre-reception); 4, preanalytical (post-reception); 5, analytical; 6, post-analytical.
